# Stimulant use disorder diagnosis and opioid agonist treatment dispensation following release from prison: a cohort study

**DOI:** 10.1186/s13011-022-00504-z

**Published:** 2022-11-24

**Authors:** Heather Palis, Bin Zhao, Pam Young, Mo Korchinski, Leigh Greiner, Tonia Nicholls, Amanda Slaunwhite

**Affiliations:** 1 BC Centre for Disease Control, University of British Columbia, 655 W 12Th Avenue, BC V5Z 4R4 Vancouver, Canada; 2 Department of Psychiatry, University of British Columbia, 2255 Wesbrook Mall, Vancouver, BC V6T 2A1 Canada; 3 Unlocking the Gates Services Society, 22838 Lougheed Hwy. Unit 104, Maple Ridge, BC V2X 2V6 Canada; 4 BC Corrections, 1001 Douglas St, Victoria, BC V8W 2C5 Canada; 5 BC Mental Health and Substance Use Services, 4949 Heather St, Vancouver, BC V5Z 3L7 Canada; 6 School of Population and Public Health, University of British Columbia, 2206 E Mall, Vancouver, BC V6T 1Z3 Canada

**Keywords:** Stimulant use disorder, Opioid use disorder, Opioid agonist treatment, Incarceration, Prison, Mental health

## Abstract

**Background:**

Concurrent opioid and stimulant use is on the rise in North America. This increasing trend of use has been observed in the general population, and among people released from prison in British Columbia (BC), who face an elevated risk of overdose post-release. Opioid agonist treatment is an effective treatment for opioid use disorder and reduces risk of overdose mortality. In the context of rising concurrent stimulant use among people with opioid use disorder, this study aims to investigate the impact of stimulant use disorder on opioid agonist treatment dispensation following release from prison in BC.

**Methods:**

Linked health and corrections records were retrieved for releases between January 1^st^ 2015 and December 29^th^ 2018 (*N* = 13,380). Hospital and primary-care administrative health records were used to identify opioid and stimulant use disorder and mental illness. Age, sex, and health region were derived from BC’s Client Roster. Incarceration data were retrieved from provincial prison records. Opioid agonist treatment data was retrieved from BC’s provincial drug dispensation database. A generalized estimating equation produced estimates for the relationship of stimulant use disorder and opioid agonist treatment dispensation within two days post-release.

**Results:**

Cases of release among people with an opioid use disorder were identified (*N* = 13,380). Approximately 25% (*N* = 3,328) of releases ended in opioid agonist treatment dispensation within two days post-release. A statistically significant interaction of stimulant use disorder and mental illness was identified. Stratified odds ratios (ORs) found that in the presence of mental illness, stimulant use disorder was associated with lower odds of obtaining OAT [(OR) = 0.73, 95% confidence interval (CI) = 0.64–0.84)] while in the absence of mental illness, this relationship did not hold [OR = 0.89, 95% CI = 0.70–1.13].

**Conclusions:**

People with mental illness and stimulant use disorder diagnoses have a lower odds of being dispensed agonist treatment post-release compared to people with mental illness alone. There is a critical need to scale up and adapt opioid agonist treatment and ancillary harm reduction, and treatment services to reach people released from prison who have concurrent stimulant use disorder and mental illness diagnoses.

**Supplementary Information:**

The online version contains supplementary material available at 10.1186/s13011-022-00504-z.

## Background

People who are incarcerated have a significantly higher health burden compared to the general population, including infectious diseases, mental illness, and substance use disorders [1–4]. The period of transition to community is a high-risk period for negative outcomes, with studies revealing mortality rates nearly 13 times higher in the weeks following release, as compared to the general population [5]. While a number of factors contribute to this high mortality in the transition from prison to community, studies have identified that opioids contribute to nearly 1 in 8 post release fatalities [6]. People with histories of incarceration are known to face an elevated risk of overdose, particularly in the days and weeks following release [7]. In British Columbia (BC) (Canada’s third most populous province) approximately 70% of people who are incarcerated have been identified as having either a mental health or substance use disorder [8], and the most commonly reported substances used at admission are opioids and stimulants [9]. Opioid agonist treatment (OAT) is available as the first line treatment for opioid use disorder (OUD) in BC, the most commonly available forms of which include methadone (a full opioid agonist) and buprenorphine (a partial opioid agonist), both of which are effective at reducing opioid craving and withdrawal, reducing illicit opioid use [10, 11] and protecting against overdose mortality [12].

OAT dispensation has increased in BC’s provincial prisons in recent years, with the proportion of people with OUD who had received OAT more than doubling from approximately 30% in 2015 to 65% in 2017 [13]. OAT has also been expanded in community settings in BC, with an increasing range of available medications [14], and new prescribing authorities assigned to nurse practitioners [15]. For people released from prison, the timeliness of OAT dispensation is critical. Research has found that reduced tolerance following release can increase risk of overdose on the day of and in the 1–2 weeks immediately following release [7]. Furthermore, withdrawal symptoms set in within 24–48 h of last OAT dose [16]; therefore, expedient continuity of medication dispensation in the community is critical to reducing overdose risk. In the absence of access to OAT, people with OUD are more likely to engage with the illicit drug supply, which is increasingly contaminated by potent illicit opioids like fentanyl [17] which have been attributed to the ongoing rise in overdose deaths in the province [18].

Alongside fentanyl, methamphetamine has also increasingly been detected among people who have died of illicit drug toxicity (overdose) in BC, and stimulant use is on the rise in North America among people who use opioids [19, 20]. This trend has also been observed among people admitted to BC’s prisons, where the prevalence of reported methamphetamine use at intake increased nearly fivefold between 2009–2017 [21]. This is particularly concerning given polysubstance use with opioids has been associated with compounded risk of adverse health effects, such as overdose [22]. Furthermore, people with concurrent OUD and stimulant use disorder (StUD) often have greater health service needs compared to people with OUD alone, yet are less likely to be engaged and retained in care [23]. Given OAT is a first line treatment for OUD, it remains a critical intervention that should be offered in a timely manner during the period of transition from prison to community for all people with OUD who want access to it. Due to the limited availability of pharmacological and psychosocial treatments for StUD [24–26], people who use stimulants face significant gaps in their substance use service needs, making people with concurrent OUD and StUD a priority for intervention. OAT can serve as an opportunity for engagement in care for this population [27, 28], who must not be further excluded from access to evidence-based health and substance use services.

Prior studies have found that stimulant use can interfere with OAT outcomes, such as reductions in illicit opioid use [29] and long-term retention [23]. While studies have investigated the impact of stimulant use on OAT retention, [23, 30] questions about the impact of StUD on OAT dispensation following release from prison have not been explored. Given the rising prevalence of stimulant use in BC among people who have been incarcerated [21], this study aims to investigate how having a history of stimulant use disorder impacts the uptake of opioid agonist treatment within two days of being released from prison in BC. Analyses will examine the impact of other covariates including age, sex, and concurrent mental illness diagnoses.

## Methods

### Study population

This study used a 20% random sample of the general population of British Columbians, contained within the British Columbia Provincial Overdose Cohort (BC-ODC) [31]. The BC-ODC brings together administrative health data and corrections data linked through BC’s Client Roster. The Roster is comprised of records for provincial health insurance which is compulsory for all residents of BC (including Canadian citizens, permanent residents, persons on visas > 6 months and their dependents).

This analysis included a cohort of people who had a record in the client roster between January 1st 2015 and December 29th 2018 with at least one release from one of BC’s ten provincial prisons in this period. OUD was determined at the time of release using a standard algorithm that includes ICD-9 and ICD-10 codes from hospital, [32] primary care, [33] (ICD9 = 304.0, 305.5; ICD10 = F11) and drug dispensation records dating back to January 1^st^ 2010. People were required to have at least one hospitalization or one primary care visit or an OAT dispensation (See Table S1).  The analyses were not pre-registered, and all results should be considered exploratory.

### Outcome measure

The outcome of interest was community OAT dispensation within two days of release from a provincial prison. This time frame reflects critical window of time (24–48 h) within which OAT dispensation is necessary post-release to avoid withdrawal and subsequent return to the illicit opioid supply, and was determined in consultation with an advisory group of people with lived and living experience of incarceration, opioid use, and OAT access. OAT dispensation was retrieved from PharmaNet (provincial drug dispensation database) records [34] and reflects medications available in BC for the treatment of OUD (See Table S2). For each release from prison, OAT dispensation within two days of release was determined to be present when there was an OAT dispensation recorded in PharmaNet on the first or second day following the date of release. PharmaNet does not distinguish between medications dispensed in the prison on the day of release vs. in community on the day of release. As such, dispensations on the day of release were not considered in the outcome.

### Exposure measure

The exposure of interest was StUD diagnosis. StUD was determined at the time of release using ICD codes from hospital (ICD9 = 304.2, 304.4, 305.6, 305.7) and primary care (ICD10 = F14,F15) records dating back to January 1^st^ 2010. The exposure was determined at the time of release for each release. As such, the exposure was time varying, and could change from one release to the next (e.g. a person with no StUD diagnosis at their first release may have one at a future release). In order to have the StUD diagnosis assigned, one hospitalization or two primary care visits with the relevant ICD9/10 code was required within 1 year of each other.

### Covariates

Variables that were hypothesized to confound the relationship between StUD diagnosis and OAT dispensations were: age (categorized), sex (female or male), health authority of residence at time of admission, number of prior incarcerations (by time of release, dating back to January 1^st^ 2015), year of release, and mental illness diagnosis prior to release (dating back to January 1^st^ 2010). Mental illness was defined based on the presence of one hospitalization record or two outpatient records within one year for anxiety, depression, schizophrenia, bipolar, personality or stress disorder (See Table S3).

### Data analysis

The characteristics of the sample are presented by the exposure (StUD) (Table 1) and outcome (OAT dispensation) (Table 2) at time of release, for each release. A generalized estimating equation (GEE) was used to estimate the odds of OAT dispensation within two days post-release among people with a history of StUD compared to people without. In the GEE, a logit link and exchangeable correlation structure was used to adjust for multiple releases for the same person [35]. Unadjusted and adjusted odds ratios with corresponding 95% confidence intervals are presented (Table 3). Multivariable models included all exposure variables outlined above, and each exposure, with the exception of sex, could vary from one release to the next.

**Table 1:** Demographic, geographic, health, and corrections characteristics of people with an opioid use disorder diagnosis released from provincial prisons between Jan 1 2015- Dec 29 2018 (*N* = 13,380 releases), by stimulant use disorder diagnosis

	**Total releases** **N(%)** ***N* = 13,380**	**Stimulant use disorder diagnosis** **N(%)** ***N* = 4963**	**No stimulant use disorder diagnosis** **N(%)** ***N* = 8417**	***P* value**
**Sex**				
Female	1625(12.15)	768(47.26)	857(52.74)	< 0.001
Male	11,755(87.85)	4195(35.69)	7560(64.31)	
**Age**				
< 30	5927 (44.30)	2151(36.29)	3776(63.71)	0.092
30–39	5155(38.53)	1981(38.43)	3174(61.57)	
40–49	1895(14.16)	684(36.09)	1211(63.91)	
> = 50	403(3.01)	147(36.48)	256(63.52)	
**Health Authority**				
Unknown	253(1.89)	5(1.98)	248(98.02)	< 0.001
Interior	1608(12.02)	550(34.20)	1058(65.80)	
Northern	824(6.16)	383(46.48)	441(53.52)	
Vancouver Coastal	3089(23.09)	1584(51.28)	1505(48.72)	
Vancouver Island	1673(12.50)	444(26.54)	1229(73.46)	
Fraser	5933(44.34)	1997(33.66)	3936(66.34)	
**Mental Illness diagnosis prior to release**				
Yes	8685 (64.91)	4248(48.91)	4437(51.09)	< 0.001
No	4695(35.09)	715(15.23)	3980(84.77)	
**Prior number of incarcerations (only back to 2015) at time of release**^a^				
0	3969(29.67)	1111(27.99)	2858(72.01)	< 0.001
1	2581(19.29)	810(31.38)	1771(68.62)	
2 +	6830(51.05)	3042(44.54)	3788(55.46)	
**Year of release**				
2015	2533(18.93)	834(32.93)	1699(67.07)	< 0.001
2016	3100(23.17)	1094(35.29)	2006(64.71)	
2017	3863(28.87)	1442(37.33)	2421(62.67)	
2018	3884(29.03)	1593(41.04)	2291(58.99)	

^a^Distribution of prior incarcerations Mean: 2.86 (SD: 3.99), Median = 2.00, [IQR = 0.00,4.00] Range:0–49). The “unknown” category for health authority represents cases for which health authrortity of residence data were missing. These records were retained in the analysis in their own “unkown” level of the health authority variable. Data are presented at the release level, not the person level

**Table 2:** Demographic, geographic, health, and corrections characteristics of people with an opioid use disorder diagnosis released from provincial prisons between Jan 1 2015- Dec 29 2018 (*N* = 13,380 releases), by OAT dispensation within two days of release

	**Total releases** **N(%)** ***N* = 13,380**	**No OAT dispensation** **N(%)** ***N* = 10,052** **75.1%**	**OAT** **dispensation N(%)** ***N* = 3,328** **24.9%**	***P* value**
**Stimulant use disorder**				
Yes	4963 (37.09)	3750(75.56)	1213(24.44)	0.375
No	8417(62.91)	6302(74.87)	2115(25.13)	
**Sex**				
Female	1625(12.15)	1177(72.43)	448(27.57)	0.007
Male	11,755(87.85)	8875(75.50)	2880(24.50)	
**Age**				
< 30	5927 (44.30)	4568(77.07)	1359(22.93)	< 0.001
30–39	5155(38.53)	3840(74.49)	1315(25.51)	
40–49	1895(14.16)	1365(72.03)	530(27.97)	
> = 50	403(3.01)	279(69.23)	124(30.77)	
**Health Authority**				
Unknown	253(1.89)	228(90.12)	25(9.88)	< 0.001
Interior	1608(12.02)	1133(70.46)	475(29.54)	
Northern	824(6.16)	650(78.88)	174(21.12)	
Vancouver Coastal	3089(23.09)	2339(75.72)	750(24.28)	
Vancouver Island	1673(12.50)	1121(67.01)	552(32.99)	
Fraser	5933(44.34)	4581(77.21)	1352(22.79)	
**Mental Illness diagnosis prior to release**				
Yes	8685 (64.91)	6394(73.62)	2291(26.38)	< 0.001
No	4695(35.09)	3658(77.91)	1037(22.09)	
**Prior number of incarcerations (only back to 2015) at time of release**^**a**^				
0	3969(29.67)	2988(75.28)	981(24.72)	0.4927
1	2581(19.29)	1959(75.90)	622(24.10)	
2 +	6830(51.05)	5105(74.74)	1725(25.26)	
**Year of release**				
2015	2533(18.93)	2086(82.35)	447(17.75)	< 0.001
2016	3100(23.17)	2420(78.06)	680(21.94)	
2017	3863(28.87)	2800(72.48)	1063(27.52)	
2018	3884(29.03)	2745(70.70)	1138(29.30)	

^a^Distribution of prior incarcerations Mean: 2.86 (SD: 3.99), Median = 2.00, [IQR = 0.00,4.00] Range:0–49). Data are presented at the release level, not the person level

**Table 3:** Unadjusted and adjusted odds ratio estimates of OAT dispensation within two days of release among people with an opioid use disorder diagnosis who were released from provincial prisons between Jan 1 2015- Dec 29 2018 (*N* = 13,380 releases)

	**Unadjusted** **OR (95% CIs)**	**Adjusted** **OR (95% CIs)**
**Stimulant use disorder**		
Yes	0.99(0.89–1.12)	**0.84(0.74–0.95)**
No	Reference	Reference
**Sex**		
Female	**1.12(0.97–1.30)**	1.16(0.99–1.35)
Male	Reference	Reference
**Age**		
< 30	**0.64(0.47–0.86)**	**0.63(0.47–0.86)**
30–39	0.75(0.56–1.01)	0.75(0.56–1.02)
40–49	0.97(0.63–1.18)	0.90(0.66–1.24)
> = 50	Reference	Reference
**Health Authority**		
Fraser	Reference	Reference
Interior	1.44(1.24–1.69)	**1.31(1.12–1.54)**
Vancouver Island	**1.70(1.45–2.00)**	**1.64(1.40–1.93)**
Northern	0.94(0.74–1.18)	0.87(0.69–1.10)
Vancouver Coastal	1.10(0.95–1.27)	1.08(0.93–1.25)
Unknown	**0.31(0.17–0.58)**	**0.35(0.17–0.67)**
**Mental Illness diagnosis prior to release**		
Yes	**1.31(1.17–1.46)**	**1.35(1.20–1.52)**
No	**Reference**	**Reference**
**Prior number of incarcerations (only back to 2015) at time of release**^**a**^		
0	**0.83(0.76–0.91)**	1.00(0.91–1.11)
1	**0.83(0.75–0.92)**	0.95(0.86–1.05)
2 +	Reference	Reference
**Year of release**		
2015	Reference	Reference
2016	**1.31(1.14–1.51)**	**1.31(1.13–1.53)**
2017	**1.83(1.59–2.12)**	**1.86(1.59–2.16)**
2018	**2.13(1.83–2.47)**	**2.15(1.82–2.52)**

^a^Distribution of prior incarcerations Mean: 2.86 (SD: 3.99), Median = 2.00, [IQR = 0.00,4.00] Range:0–49) Unknown health authority = No fixed address. In adjusted analysis, the estimate for each covariate is adjusted for all other covariates in the model. So, the estimate for Stimulant use disorder in the adjusted column, is after adjusting for sex, age, health authority, mental illness, prior number of incarcerations, and year of release

Estimates from GEE models with exchangeable correlation structure- accounting for repeated observations arising from the same person

In the adjusted GEE model, StUD was associated with OAT dispensation only when mental illness was adjusted for. As such, in post-hoc analyses, an interaction term between StUD diagnosis and mental illness was tested and was statistically significant. One categorical variable was created to reflect all four levels of the 2 × 2 interaction (i.e. presenting the risk factor of interest (StUD, yes vs. no) within both levels of the suspected effect modifying variable (mental illness yes and no) (Table 4). This approach produced stratified ORs, with estimates for each stratum with one single reference category, allowing for the interaction to be estimated on an additive scale. This is a necessary approach for the determination of the separate impact of each level of both variables on the outcome [36]. In the final model, the Bonferroni correction was used to adjust for multiple comparisons [37] (See Table S9). In sensitivity analyses, the models were rerun with different variations of the OAT outcome to test whether the associations identified held when using different outcome definitions (i.e. dispensation within 1,3,7 days, and within 2 days, including the day of release) (See Tables S4-S8). Sensitivity analyses were also conducted among a reduced sample who had an OUD diagnosis in the 1 year prior to their release (*N* = 5,959), and to determine whether the interaction term held over time (by year of release). The only variable with missing data was health authority. These records were classified as “Unknown” and were included in the analysis as their own level of the health authority variable. All analyses were conducted in SAS Enterprise Guide.

**Table 4:** Mental illness and stimulant use disorder stratum specific unadjusted and adjusted Odds ratio estimates of OAT dispensation within two days of release among people with an opioid use disorder diagnosis who were released from provincial prisons between Jan 1 2015- Dec 29 2018 (*N* = 13,380 releases)

	**Unadjusted** **OR (95% CIs)**	**Adjusted** **OR (95% CIs)**
**No mental illness**		
No stimulant use disorder	**0.66(0.58–0.76)**	**0.64(0.57–0.74)**
Stimulant use disorder	0.93(0.77–1.18)	0.89(0.70–1.13)
**Mental illness**		
No stimulant use disorder	Reference	Reference
Stimulant use disorder	**0.81(0.70–0.92)**	**0.73(0.64–0.84)**
**Sex**		
Female	**1.12(0.97–1.30)**	1.16(0.99–1.35)
Male	Reference	Reference
**Age**		
< 30	**0.64(0.47–0.86)**	**0.63(0.47–0.86)**
30–39	0.75(0.56–1.01)	0.75(0.56–1.02)
40–49	0.97(0.63–1.18)	0.90(0.66–1.24)
> = 50	Reference	Reference
**Health Authority**		
Fraser	Reference	Reference
Interior	1.44(1.24–1.69)	**1.31(1.12–1.54)**
Vancouver Island	**1.70(1.45–2.00)**	**1.64(1.40–1.93)**
Northern	0.94(0.74–1.18)	0.87(0.69–1.10)
Vancouver Coastal	1.10(0.95–1.27)	1.08(0.93–1.25)
Unknown	**0.31(0.17–0.58)**	**0.35(0.17–0.67)**
**Prior number of incarcerations (only back to 2015) at time of release(a)**		
0	**0.83(0.76–0.91)**	1.00(0.91–1.11)
1	**0.83(0.75–0.92)**	0.95(0.86–1.05)
2 +	Reference	Reference
**Year of release**		
2015	Reference	Reference
2016	**1.31(1.14–1.51)**	**1.31(1.13–1.53)**
2017	**1.83(1.59–2.12)**	**1.86(1.59–2.16)**
2018	**2.13(1.83–2.47)**	**2.15(1.82–2.52)**

In adjusted analysis, the estimate for each covariate is adjusted for all other covariates in the model. So, in the adjusted column, the estimate for each of the levels of the mental illness by stimulant use disorder interaction are adjusted for sex, age, health authority, mental illness, prior number of incarcerations, and year of release

## Results

Among the 1,089,677 people in the 20% random sample, there were 17,930 cases of release from prison during the study period. Of these, 14,663 had an OUD diagnosis prior to their release. An additional 1,278 releases were excluded. Of these 759(59.4%) were excluded due to intermittent sentence (sentence of < 90 days served in community under conditions of parole, and with some time (usually weekends) spent in custody). The remaining 40.6% (*N* = 519) of excluded records were excluded because the incarceration event lasted < 1 day. The final sample included 13,380 cases of release from provincial prisons between January 1^st^ 2015 and December 29^th^ 2018 among people with an OUD diagnosis. Of these, 37.1% (*N* = 4,963) had a concurrent StUD diagnosis.

Females were more likely than males to have a StUD diagnosis. Nearly half of people with mental illness had a StUD diagnosis, while only approximately 15% of people without mental illness had StUD diagnosis. There were substantial regional variations in StUD diagnosis across the province, for example with approximately half of people in Vancouver Coastal region (largest urban centre in BC) and Northern region (largest rural centre) having a StUD diagnosis, and with lower proportions in the other regions of the province. Cases of release with more prior releases were more common in people with StUD diagnoses, for example, people with StUD made up approximately 30% of cases of release with 0 or 1 prior incarceration, and 45% of cases with 2 or more prior incarcerations. StUD diagnosis was increasing over time, representing approximately one-third of releases in 2015, and more than 41% of releases in 2018 (See Table 1).

Of the 13,380 cases of release, approximately 25% (*N* = 3,328) ended in OAT dispensation within 2 days of release. People with StUD were no less likely than people without StUD to be dispensed OAT (24.4% vs 25.1%, *p* = 0.375). OAT dispensation within 2 days was more likely among females compared to males (27.6% vs 24.5%, *p* = 0.007), among older compared to younger people (30.8% among >  = 50 vs. 22.9% among < 30, *p* =  < 0.001), and was less likely among people with mental illness compared to those without (26.4% vs. 22.1%, *p* < 0.001). OAT dispensation within two days of release has increased over time, with 17.8% of people accessing in 2015 compared to 29.3% in 2018. In the GEE analysis, StUD diagnosis was not associated with the outcome of OAT dispensation in unadjusted analyses, however it was in the adjusted analyses (OR(95%CI): 0.84(0.74–0.95), *p* = 0.006). An interaction term of StUD and mental illness and was found to be statistically significant. The GEE model was repeated adjusting for the 2 by 2 interaction of StUD and mental illness. The stratified odds ratios (with separate indicators for StUD in the presence and absence of mental illness) revealed that in the presence of mental illness, StUD was associated with lower odds of OAT dispensation (0.73(0.64–0.84), *p* =  < 0.001), while in the absence of mental illness, this relationship did not hold (0.89(0.70–1.13), *p* = 0.344).

In the adjusted analyses, the odds of OAT dispensation increased with age. The youngest age group (< 30 years) had lower odds of OAT dispensation (0.63(0.47–0.86), *p* = 0.003) compared to the oldest age group (> = 50 years). The odds of OAT dispensation were higher in Interior Health (1.31(1.12–1.54), *p* =  < 0.001) and Vancouver Island Health (1.64(1.40–1.93), *p* < 0.001)) relative to Fraser Health (the region where the majority of releases occur). The odds of OAT dispensation increased each year relative to 2015, and reached more than twice the odds of dispensation in 2018 (vs 2015) (2.15(1.82–2.52), *p* =  < 0.001). In sensitivity analyses, the analysis was repeated using multiple variations of the OAT outcome and in all analyses, the main findings held, where StUD was associated with lower odds of OAT dispensation, only in the presence of concurrent mental illness. Among people with an OUD diagnosis in the one year prior to release (*N* = 5,959) a similar proportion of people accessed OAT within two days (*N* = 1454, 24.4%), as was found in the overall sample (*N* = 3328, 24.9%) in Table 2. The interaction term was tested by year and was found to be statistically significant in all years, with the exception of 2015 (*p* = 0.467). This could be driven by 2015 having a smaller number of releases compared to all other years, and having the lowest proportion of stimulant use disorder diagnoses and OAT access among releases, as compared to all other years.

## Discussion

In this population-based study of people with OUD who were released from provincial prisons, we found that in unadjusted analyses, StUD diagnosis was not associated with a reduced odds of OAT dispensation in the two days following release. However, people with concurrent mental illness and StUD had lower odds of OAT dispensation compared to the group with mental illness alone. This suggests that among people released from BC’s prisons, people who experience concurrent health and substance use challenges alongside OUD are less likely to receive OAT post-release.

Prior studies have drawn connections between stimulant use and mental illness. For example, systematic reviews report that psychoses is highest among people who use methamphetamine or cocaine frequently and who have diagnosed dependence on these substance [38, 39]. However, the relationship between stimulant use and mental illness is complex, and is affected by a number of factors which vary from one person to the next, making it is difficult to draw directional or causal conclusions about this relationship. Nevertheless, stimulant use is on the rise in North America. For example, a study in the United States found that methamphetamine use nearly doubled between 2011–2017, from 18.8% to 34.2% among people with OUD [19]. In our study, StUD diagnosis increased among people released from prison, from 32% in 2015 to 41% in 2018. This aligns with population-level analyses of drug toxicology data in BC, where methamphetamine has been detected in approximately 40% of overdose deaths [18]. As such, the population with OUD and StUD is growing, and the health and substance use service needs of this population require increased attention. This may necessitate ongoing education for care providers [40], given people who use stimulants such as methamphetamine have been known to face stigma in their health care encounters [41–43] which can be compounded by concurrent mental illness [44].

In this study, we found the odds of OAT dispensation were lowest in people aged < 30, who were approximately 35% less likely to be dispensed OAT within two days of release, compared to people aged >  = 50. People aged < 30 made up the highest proportion of releases in the sample (44.2%) and therefore represent an important group whose substance use service needs must be prioritized. Prior studies have revealed that young people might be less likely to come in contact with care due to unique fears not facing adults, including concerns about involuntary detention, or disclosure of their substance use to family members [45, 46]. Youth are known to encounter stigma in seeking OAT [47] and OAT dispensations are lower in youth compared to adults [48]. In addition to efforts to expand accessibility of OAT for youth, youth advocates have recently called for a movement away from abstinence-based approaches, and ensuring the availability of confidential, peer-led interventions [49]. These services can engage youth in harm reduction services, and may serve as a path to treatment if or when youth are ready to engage in these services.

In this analysis, we found that the odds of OAT dispensation within 2 days post-release increased over time, from 17.8% in 2015 to 29.3% in 2018. This translated to cases of release in 2018 having more than twice the odds of OAT dispensation compared to releases in 2015. This is consistent with research on OAT inside provincial prisons which found that the proportion of people receiving OAT in provincial prison in BC doubled between 2015–17 [13]. This suggests a high level of service need when transitioned to community, however we found only about 25% of people released between 2015–18 were dispensed OAT within two days of their release. This two-day window of access is particularly important given, evidence suggests withdrawal will set in within 24–48 h of last OAT dose [16]. In cases of withdrawal without access to OAT, people are more likely to return to illicit substance use [50]. Our team’s prior studies have found that the day of release as a time of elevated risk for overdose [7], further suggesting the critical need for timely OAT dispensation post-release. While nearly one third of people with OUD released from prison have access to OAT in the two days post-release, two thirds of people do not. There is a significant need for expanded interventions to reach and meet people with OUD who want access to OAT.

Efforts have been made in BC to improve the accessibility of OAT in community for people released from prison and to promote continuity of OAT prescriptions from prison to community. For example, nurses in BC’s prisons can communicate with community pharmacies and physicians to ensure they have access to complete community medical records and to bridge connections to OAT prescriptions upon release [51]. For people facing concurrent mental illness and StUD alongside their OUD, additional low-barrier, targeted support must be provided. Given the known stigma facing people with histories of incarceration and known distrust of the health care system in this population [52], peer-led outreach services can play a critical role in reducing barriers to service engagement. Peer outreach workers are people who have lived experience of the same challenges facing the population, including incarceration, substance use, and/or mental health diagnoses and play an instrumental role in developing rapport with people who may need services [53]. Prior studies have found the peer model is an effective outreach model to engage people who use substances with care [54]. Existing peer-led programs for people released from prison such as Unlocking the Gates Services Society [55, 56] must be supported to expand their reach, to engage people, including those with StUD and mental illness who may face the most barriers to service access.

While OAT is effective at reducing illicit opioid use, it has also been used to connect people to other health services. For example, prior studies have found that OAT prescribers are well positioned to offer care for HCV, where prescribed medications can be effectively incorporated into OAT care [57]. Furthermore, in some OAT clinics, physicians prescribe psychostimulants to people who use cocaine and or methamphetamine to help reduce cravings, withdrawal and to support reduced illicit stimulant use [58–60]. While this practice has remained relatively limited, a recent systematic review has highlighted the effectiveness of psychostimulants in reducing illicit stimulant use [61] and studies have shown that psychostimulant prescribing alongside OAT can promote adherence to both medications [28] and improve psychosocial outcomes [62]. Given the growing proportion of people released from prisons in BC who have StUD diagnoses, these medications could be prescribed alongside OAT to support reductions in illicit stimulant use [63].

In community, and in prison, diversified OAT options are needed to engage a wide range of people who use drugs, including people with mental illness and StUD who we have found are less likely to receive timely OAT dispensation post-release. It is possible that they have preferences for treatment, (e.g. different medications or routes of administration such as injecting or smoking) [64] that are not currently available. As such, future research must focus on examining the service preferences of people with OUD who have concurrent StUD and mental illness. Furthermore, not everyone is ready to engage in treatment, nor abstinence, and a safer supply [65–67] of alternatives to the illicit drug supply must be available to keep people alive in the context of an unregulated and unsafe illicit drug supply.

There are a number of limitations of the present study to be considered. First, we use ICD9/10 codes to define OUD and StUD. As such people who use opioids and stimulants but who have not contacted health services for care are not captured. Furthermore, the definitions of OUD and StUD rely on historical administrative health records, and do not confirm that participants met criteria for these diagnoses at the exact time of their release from incarceration. We focus on OAT dispensation within two days following release, but do not examine subsequent treatment engagement or long-term retention which could be examined in future studies in this population. Furthermore, we have not investigated the impact of stimulant use disorder diagnosis on OAT access while incarcerated, nor its impact on the continuity of OAT access between correctional settings and community. Future studies could consider the characteristics of OAT while incarcerated (e.g. dose, duration of access, timeliness of access) on continuity of OAT post-release for people with and without concurrent SUDs such as stimulant use disorder. We report on biological sex as a binary variable (male vs. female) as data on gender identity is not available.

## Conclusions

Access to health and substance use services in the days immediately following release from prison is critical to reducing negative outcomes like overdose and all-cause mortality. For people with OUD, OAT remains an effective evidence-based intervention. This study demonstrates that people with mental illness and StUD have reduced odds of obtaining OAT post-release. In the context of an ongoing overdose crisis that disproportionally impacts people released from prison, there is a critical need to scale up and adapt OAT and ancillary peer support, harm reduction, treatment, and health services in order to reach people with concurrent StUD and mental illness.

## Supplementary Information

**Additional file 1: Table S1.** Exposure: type of substance use disorder diagnosis. **Table S2.** Outcome: Medications and drug identification numbers (DINs) included in definition of opioid agonist treatment. **Table S3.** Mental Illness Algorithm. **Table S4.** Demographic, geographic, and health and corrections characteristics of people with an opioid use disorder diagnosis released from provincial correctional centres between Jan 1 2015- Dec 29 2018 (*N*=13,380), by OAT dispensation within 1 day of release (not including day of release). **Table S5.** Demographic, geographic, and health and corrections characteristics of people with an opioid use disorder diagnosis released from provincial correctional centres between Jan 1 2015- Dec 28 2018 (*N*=13,375), by OAT dispensation within 3 days of release (not including day of release). **Table S6.** Demographic, geographic, and health and corrections characteristics of people with an opioid use disorder diagnosis released from provincial correctional centres between Jan 1 2015- Dec 24 2018 (*N*=13,353), by OAT dispensation within 7 days of release (not including day of release). **Table S7.** Demographic, geographic, and health and corrections characteristics of people with an opioid use disorder diagnosis released from provincial correctional centres between Jan 1 2015- Dec 29 2018 (*N*=13,380), by OAT dispensation within two days of release (including day of release). **Table S8.** Unadjusted and adjusted odds ratio estimates of OAT dispensation for four levels of the main exposure of interest (mental illness and stimulant use disorder diagnoses) on various OAT outcome definitions. **Table S9.** Mental illness and stimulant use disorder stratum specific unadjusted and adjusted Odds ratio estimates of OAT dispensation within two days of release among people with an opioid use disorder diagnosis who released from provincial prisons between Jan 1 2015- Dec 29 2018 (*N*=13,380) (P Value column included).

## Data Availability

The data that support the findings of this study are available through the BC Overdose Cohort but restrictions apply to the availability of these data, which were used under license for the current study, and so are not publicly available. Data are however available from the authors upon reasonable request and with permission of PopData BC.

## Abbreviations

BC: British Columbia
GEE: Generalized Estimating Equation
OAT: Opioid Agonist Treatment
OUD: Opioid Use Disorder
OR: Odds Ratio
StUD: Stimulant use disorder
