# Supplementary material for: Stimulant use disorder diagnosis and opioid agonist treatment dispensation following release from prison: a cohort study
Source: Subst Abuse Treat Prev Policy. 2022 Nov 24;17:77. doi: 10.1186/s13011-022-00504-z (PMC9694574; doi:10.1186/s13011-022-00504-z)
Supplement: Supplementary file 1 — Additional file 1: Table S1. Exposure: type of substance use disorder diagnosis. Table S2. Outcome: Medications and drug identification numbers (DINs) included in definition of opioid agonist treatment. Table S3. Mental Illness Algorithm. Table S4. Demographic, geographic, and health and corrections characteristics of people with an opioid use disorder diagnosis released from provincial correctional centres between Jan 1 2015- Dec 29 2018 (N=13,380), by OAT dispensation within 1 day of release (not including day of release). Table S5. Demographic, geographic, and health and corrections characteristics of people with an opioid use disorder diagnosis released from provincial correctional centres between Jan 1 2015- Dec 28 2018 (N=13,375), by OAT dispensation within 3 days of release (not including day of release). Table S6. Demographic, geographic, and health and corrections characteristics of people with an opioid use disorder diagnosis released from provincial correctional centres between Jan 1 2015- Dec 24 2018 (N=13,353), by OAT dispensation within 7 days of release (not including day of release). Table S7. Demographic, geographic, and health and corrections characteristics of people with an opioid use disorder diagnosis released from provincial correctional centres between Jan 1 2015- Dec 29 2018 (N=13,380), by OAT dispensation within two days of release (including day of release). Table S8. Unadjusted and adjusted odds ratio estimates of OAT dispensation for four levels of the main exposure of interest (mental illness and stimulant use disorder diagnoses) on various OAT outcome definitions. Table S9. Mental illness and stimulant use disorder stratum specific unadjusted and adjusted Odds ratio estimates of OAT dispensation within two days of release among people with an opioid use disorder diagnosis who released from provincial prisons between Jan 1 2015- Dec 29 2018 (N=13,380) (P Value column included). [file 13011_2022_504_MOESM1_ESM.docx]

**Supplement**

**Table S1: Exposure: type of substance use disorder diagnosis**

| Diagnosis | ICD9 codes | ICD10 codes | Algorithm |
| --- | --- | --- | --- |
| **Substance use disorder** | | | |
| Stimulant use disorder | 3042, 3052, 3044, 3057 | F14, F15 | Either: 2 family physician visits or 1 hospitalization in 1 year |
| Opioid use disorder | 3040, 3055 | F11 | Either: 1 family physician visit or 1 hospitalization or an OAT dispensation. |

**Footnote:** Algorithms use data going back to January 1 2010.

**Table S2: Outcome: Medications and drug identification numbers (DINs) included in definition of opioid agonist treatment**

| **Medication name** | **Medication DIN PINs** |
| --- | --- |
| Methadone, buprenorphine, buprenorphine/naloxone, slow release oral morphine, diacetylmorphine | \| 999792 \| \| --- \| \| 66999990 \| \| 66999991 \| \| 999793 \| \| 66999992 \| \| 66999993 \| \| 22123349 \| \| 22123346 \| \| 67000012 \| \| 67000009 \| \| 67000010 \| \| 67000011 \| \| 67000008 \| \| 67000007 \| \| 67000005 \| \| 67000006 \| \| 66999997 \| \| 66999998 \| \| 66999999 \| \| 67000000 \| \| 67000001 \| \| 67000002 \| \| 67000003 \| \| 67000004 \| \| 22123347 \| \| 2242963 \| \| 2295695 \| \| 2408090 \| \| 2424851 \| \| 2453908 \| \| 655619 \| \| 781460 \| \| 22123348 \| \| 2242964 \| \| 2295709 \| \| 2408104 \| \| 2424878 \| \| 2453916 \| \| 781479 \| \| 655627 \| \| 2468085 \| \| 2468093 \| \| 2474921 \| \| 999776 \| |

**Table S3: Mental Illness Algorithm**

| Diagnosis | ICD9 codes | ICD10 codes | Algorithm |
| --- | --- | --- | --- |
| **Mental illness** | | | |
| Depression | 300.4  311  50B (also requires another code to qualify) | F32  F33  F34.1 | Either: 2 family physician visits or 1 hospitalization in 1 year |
| Anxiety | 300 (excluding 300.4) 50B (also requires another code to qualify) | F40  F41 | Either: 2 family physician visits or 1 hospitalization in 1 year |
| Stress and adjustment disorder | 308 309 | F43 | Either: 2 family physician visits or 1 hospitalization in 1 year |
| Schizophrenia | 295  297  298 | F20  F21  F22  F23  F24  F25  F28  F29 | Either: 2 family physician visits or 1 hospitalization in 1 year |
| Bipolar disorder | 296 | F30 F31 F34 (excluding F34.1) F38 F39 | Either: 2 family physician visits or 1 hospitalization in 1 year |

**Table S4: Demographic, geographic, and health and corrections characteristics of people with an opioid use disorder diagnosis released from provincial correctional centres between Jan 1 2015- Dec 29 2018 (N=13,380), by OAT dispensation within 1 day of release (not including day of release)**

|  | **Total**  **N(%)**  **N= 13,380** | **No OAT dispensation**  **N(%)**  **N=10,753**  **80.4%** | **OAT**  **dispensation N(%)**  **N=2627**  **19.6%** | **P value** |
| --- | --- | --- | --- | --- |
|  |  |  |  |  |
| **Stimulant use disorder** |  |  |  |  |
| Yes | 4963 (37.09) | 4022(81.04) | 941(18.96) | 0.1321 |
| No | 8417(62.91) | 6731(79.97) | 1686(20.03) |  |
| **Sex** |  |  |  |  |
| Female | 1625(12.15) | 1257(77.35) | 368(22.65) | 0.001 |
| Male | 11755(87.85) | 9496(80.78) | 2259(19.22) |  |
| **Age** |  |  |  |  |
| <30 | 5927 (44.30) | 4821(81.51) | 1096(18.49) | 0.0009 |
| 30-39 | 5155(38.53) | 4133(80.17) | 1022(19.83) |  |
| 40-49 | 1895(14.16) | 1486(78.42) | 409(21.58) |  |
| >=50 | 403(3.01) | 303(75.19) | 100(24.81) |  |
| **Health Authority** |  |  |  |  |
| Unknown | 253(1.89) | 231(91.30) | 22(8.70) |  |
| Interior | 1608(12.02) | 1222(76.00) | 386(24.00) | <0.001 |
| Northern | 824(6.16) | 686(83.25) | 138(16.75) |  |
| Vancouver Coastal | 3089(23.09) | 2517(81.48) | 572(18.52) |  |
| Vancouver Island | 1673(12.50) | 1226(73.28) | 447(26.72) |  |
| Fraser | 5933(44.34) | 4871(82.10) | 1062(17.90) |  |
| **Mental Illness diagnosis prior to release** |  |  |  |  |
| Yes | 8685 (64.91) | 6866(79.06) | 1819(20.94) | <0.001 |
| No | 4695(35.09) | 3887(82.79) | 808(17.21) |  |
| **Prior number of incarcerations (only back to 2015) at time of release** |  |  |  |  |
| 0 | 3969(29.67) | 3159(79.59) | 810(20.41) | 0.3316 |
| 1 | 2581(19.29) | 2087(80.86) | 494(19.14) |  |
| 2+ | 6830(51.05) | 5507(80.63) | 1323(19.37) |  |
| **Year of release** |  |  |  |  |
| 2015 | 2533(18.93) | 2170(85.67) | 363(14.33) | <0.001 |
| 2016 | 3100(23.17) | 2565(82.74) | 535(17.26) |  |
| 2017 | 3863(28.87) | 3032(78.49) | 831(21.51) |  |
| 2018 | 3884(29.03) | 2986(76.88) | 898(23.12) |  |

**Table S5: Demographic, geographic, and health and corrections characteristics of people with an opioid use disorder diagnosis released from provincial correctional centres between Jan 1 2015- Dec 28 2018 (N=13,375), by OAT dispensation within 3 days of release (not including day of release)**

|  | **Total**  **N(%)**  **N= 13,375** | **No OAT dispensation**  **N(%)**  **N=9703**  **72.5%** | **OAT**  **dispensation N(%)**  **N= 3,672**  **27.5%** | **P value** |
| --- | --- | --- | --- | --- |
|  |  |  |  |  |
| **Stimulant use disorder** |  |  |  |  |
| Yes | 4960 (37.08) | 3601(72.60) | 1359(27.40) | 0.9128 |
| No | 8415(62.91) | 6102(72.51) | 2313(27.49) |  |
| **Sex** |  |  |  |  |
| Female | 1625 (12.15) | 1143(70.34) | 482(29.66) | 0.034 |
| Male | 11750(87.85) | 8560(72.85) | 3190(27.15) |  |
| **Age** |  |  |  |  |
| <30 | 5925 (44.29) | 4423(74.65) | 1502(25.35) | <0.001 |
| 30-39 | 5153(38.52) | 3705(71.90) | 1448(28.10) |  |
| 40-49 | 1894(14.16) | 1311(69.22) | 583(30.78) |  |
| >=50 | 403(3.01) | 264(65.51) | 139(34.49) |  |
| **Health Authority** |  |  |  |  |
| Interior | 1608(12.02) | 1905(68.10) | 513(31.90) | <0.001 |
| Fraser | 5931(44.34) | 4430(74.69) | 1501(25.31) |  |
| Vancouver Coastal | 3087(23.08) | 2238(72.50) | 849(27.50) |  |
| Vancouver Island | 1672(12.50) | 1080(64.59) | 592(35.41) |  |
| Northern | 824(6.16) | 635(77.06) | 189(22.94) |  |
| Unknown | 253(1.89) | 225(88.93) | 28(11.07) |  |
| **Mental Illness diagnosis prior to release** |  |  |  |  |
| Yes | 8682(64.91) | 6140(70.72) | 2542(29.28) | <0.001 |
| No | 4693(35.09) | 3563(75.92) | 1130(24.08) |  |
| **Prior number of incarcerations (only back to 2015) at time of release** |  |  |  |  |
| 0 | 3968 (29.67) | 2904(73.19) | 1064(26.81) | 0.1385 |
| 1 | 2580(19.29) | 1897(73.53) | 683(26.47) |  |
| 2+ | 6827(51.04) | 4902(71.80) | 1925(28.20) |  |
| **Year of release** |  |  |  |  |
| 2015 | 2533(18.93) | 2038(80.46) | 495(19.54) | <0.001 |
| 2016 | 3100(23.18) | 2337(75.39) | 763(24.61) |  |
| 2017 | 3863(28.89) | 2700(69.89) | 1163(30.11) |  |
| 2018 | 3879(29.00) | 2628(67.75) | 1251(32.25) |  |

**Footnote**: Follow-up period reduced to Dec 28 2018 to allow 3 days of follow-up in dataset (to Dec 31 2018) following date of release.

**Table S6: Demographic, geographic, and health and corrections characteristics of people with an opioid use disorder diagnosis released from provincial correctional centres between Jan 1 2015- Dec 24 2018 (N=13,353), by OAT dispensation within 7 days of release (not including day of release)**

|  | **Total**  **N(%)**  **N= 13,353** | **No OAT dispensation**  **N(%)**  **N=9,031**  **67.6%** | **OAT**  **dispensation N(%)**  **N=4,322**  **32.4%** | **P value** |
| --- | --- | --- | --- | --- |
|  |  |  |  |  |
| **Stimulant use disorder** |  |  |  |  |
| Yes | 4950 (37.07) | 3326(67.19) | 1624(32.81) | 0.4034 |
| No | 8403(62.92) | 5705(67.89) | 2698(32.11) |  |
| **Sex** |  |  |  |  |
| Female | 1624(12.16) | 1069(65.83) | 555(34.17) | 0.097 |
| Male | 11729(87.83) | 7962(67.88) | 3767(32.12) |  |
| **Age** |  |  |  |  |
| <30 | 5915(44.29) | 4132(69.86) | 1783(30.14) | <0.001 |
| 30-39 | 5145(38.53) | 3442(66.90) | 1703(33.10) |  |
| 40-49 | 1890(14.15) | 1212(64.13) | 678(35.87) |  |
| >=50 | 403(3.02) | 245(60.79) | 158(39.21) |  |
| **Health Authority** |  |  |  |  |
| Interior | 1606 (12.03) | 1006(62.64) | 600(37.36) | <0.001 |
| Fraser | 5924(44.36) | 4131(69.73) | 1793(30.27) |  |
| Vancouver Coastal | 3077(23.04) | 2053(66.72) | 1024(33.28) |  |
| Vancouver Island | 1671(12.51) | 1021(61.10) | 650(38.90) |  |
| Northern | 823(6.16) | 600(72.90) | 223(27.10) |  |
| Unknown | 252(1.89) | 220(87.30) | 32(12.70) |  |
| **Mental Illness diagnosis prior to release** |  |  |  |  |
| Yes | 8667 (64.91) | 5657(65.27) | 3010(34.73) | <0.001 |
| No | 4686(35.09) | 3374(72.00) | 1312(28.00) |  |
| **Prior number of incarcerations (only back to 2015) at time of release** |  |  |  |  |
| 0 | 3964 (29.69) | 2722(68.67) | 1242(31.33) | 0.0204 |
| 1 | 2577(19.30) | 1777(68.96) | 800(31.04) |  |
| 2+ | 6812(51.01) | 4532(66.53) | 2280(33.47) |  |
| **Year of release** |  |  |  |  |
| 2015 | 2533 (18.97) | 1925(76.00) | 608(24.00) | <0.001 |
| 2016 | 3100(23.22) | 2173(70.10) | 927(29.90) |  |
| 2017 | 3863(28.92) | 2517(65.16) | 1346(34.84) |  |
| 2018 | 3857(28.89) | 2416(62.64) | 1441(37.36) |  |

**Footnote:** Follow-up period reduced to Dec 24 2018 to allow 7 days of follow-up in dataset (to Dec 31 2018) following date of release.

**Table S7: Demographic, geographic, and health and corrections characteristics of people with an opioid use disorder diagnosis released from provincial correctional centres between Jan 1 2015- Dec 29 2018 (N=13,380), by OAT dispensation within two days of release (including day of release)**

|  | **Total**  **N(%)**  **N= 13,380** | **No OAT dispensation**  **N(%)**  **N=8,675**  **64.8%** | **OAT**  **dispensation N(%)**  **N=4,705**  **35.2%** | **P value** |
| --- | --- | --- | --- | --- |
|  |  |  |  |  |
| **Stimulant use disorder** |  |  |  |  |
| Yes | 4963 (37.09) | 3177 (64.01) | 1786(35.99) | 0.1263 |
| No | 8417(62.91) | 5498(65.32) | 2919(34.68) |  |
| **Sex** |  |  |  |  |
| Female | 1625(12.15) | 968(59.57) | 657(40.43) | <0.001 |
| Male | 11755(87.85) | 7707(65.56) | 4048(34.44) |  |
| **Age** |  |  |  |  |
| <30 | 5927 (44.30) | 3983(67.20) | 1944(32.80) | <0.001 |
| 30-39 | 5155(38.53) | 3279(63.61) | 1876(36.39) |  |
| 40-49 | 1895(14.16) | 1182(62.37) | 713(37.63) |  |
| >=50 | 403(3.01) | 231(57.32) | 172(42.68) |  |
| **Health Authority** |  |  |  |  |
| Unknown | 253(1.89) | 216(85.38) | 37(14.62) |  |
| Interior | 1608(12.02) | 976(60.70) | 632(39.30) | <0.001 |
| Northern | 824(6.16) | 591(71.72) | 233(28.28) |  |
| Vancouver Coastal | 3089(23.09) | 1984(64.23) | 1105(35.77) |  |
| Vancouver Island | 1673(12.50) | 988(59.06) | 685(40.94) |  |
| Fraser | 5933(44.34) | 3920(66.07) | 2013(33.93) |  |
| **Mental Illness diagnosis prior to release** |  |  |  |  |
| Yes | 8685 (64.91) | 5404(62.22) | 3281(37.78) | <0.001 |
| No | 4695(35.09) | 3271(69.67) | 1424(30.33) |  |
| **Prior number of incarcerations (only back to 2015) at time of release** |  |  |  |  |
| 0 | 3969(29.67) | 2656(66.92) | 1313(33.08) | <0.001 |
| 1 | 2581(19.29) | 1724(66.80) | 857(33.20) |  |
| 2+ | 6830(51.05) | 4295(62.88) | 2535(37.12) |  |
| **Year of release** |  |  |  |  |
| 2015 | 2533(18.93) | 1909(75.37) | 624(24.63) | <0.001 |
| 2016 | 3100(23.17) | 2161(69.71) | 939(30.29) |  |
| 2017 | 3863(28.87) | 2406(62.28) | 1457(37.72) |  |
| 2018 | 3884(29.03) | 2199(56.62) | 1685(43.38) |  |

**Table S8: Unadjusted and adjusted odds ratio estimates of OAT dispensation for four levels of the main exposure of interest (mental illness and stimulant use disorder diagnoses) on various OAT outcome definitions**

|  | **Unadjusted**  **OR (95% CIs)** | **P value** | **Adjusted**  **OR (95% CIs)** | **P value** |
| --- | --- | --- | --- | --- |
| OAT dispensation within 2 days, not including day of release (Main result presented in manuscript) | | | | |
| **No mental illness** |  |  |  |  |
| No stimulant use disorder | **0.66(0.58-0.76)** | **<0.001** | **0.64(0.57-0.74)** | **<0.001** |
| Stimulant use disorder | 0.93(0.77-1.18) | 0.533 | 0.89(0.70-1.13) | 0.344 |
| **Mental illness** |  |  |  |  |
| No stimulant use disorder | Reference | Reference | Reference | Reference |
| Stimulant use disorder | **0.81(0.70-0.92)** | **<0.001** | **0.73(0.64-0.84)** | **<0.001** |
| OAT dispensation within 1 day, not including day of release | | | | |
| **No mental illness** |  |  |  |  |
| No stimulant use disorder | **0.66(0.57-0.76)** | **<0.001** | **0.64(0.56-0.73)** | **<0.001** |
| Stimulant use disorder | 0.83(0.65-1.08) | 0.1615 | 0.80(0.62-1.04) | 0.097 |
| **Mental illness** |  |  |  |  |
| No stimulant use disorder | Reference | Reference | Reference | Reference |
| Stimulant use disorder | **0.79(0.70-0.91)** | **0.007** | **0.74(0.64-0.84)** | **<0.001** |
| OAT dispensation within 3 days, not including day of release | | | | |
| **No mental illness** |  |  |  |  |
| No stimulant use disorder | **0.65(0.57-0.74)** | **<0.001** | **0.64(0.56-0.72)** | **<0.001** |
| Stimulant use disorder | 0.91(0.72-1.15) | 0.431 | 0.87(0.69-1.10) | 0.258 |
| **Mental illness** |  |  |  |  |
| No stimulant use disorder | Reference | Reference | Reference | Reference |
| Stimulant use disorder | **0.64(0.73-0.95)** | **0.007** | **0.75(0.65-0.85)** | **<0.001** |
| OAT dispensation within 7 days, not including day of release | | | | |
| **No mental illness** |  |  |  |  |
| No stimulant use disorder | **0.61(0.54-0.70)** | **<0.001** | **0.61(0.54-0.69)** | **<0.001** |
| Stimulant use disorder | 0.93(0.74-1.17) | 0.5330 | 0.90(0.71-1.12) | 0.3369 |
| **Mental illness** |  |  |  |  |
| No stimulant use disorder | Reference | Reference | Reference | Reference |
| Stimulant use disorder | **0.86(0.75-0.97)** | **0.017** | **0.76(0.67-0.87)** | **<0.001** |
| OAT dispensation within 2 days, including day of release | | | | |
| **No mental illness** |  |  |  |  |
| No stimulant use disorder | **0.61(0.54-0.70)** | **<0.001** | **0.62(0.54-00.71)** | **<0.001** |
| Stimulant use disorder | 0.89(0.71-1.13) | 0.3429 | 0.84(0.67-1.05) | 0.136 |
| **Mental illness** |  |  |  |  |
| No stimulant use disorder | Reference | Reference | Reference | Reference |
| Stimulant use disorder | **0.89(0.78-1.01)** | **0.071** | **0.75(0.66-0.86)** | **<0.001** |

**Footnote**: The same pattern holds across all variations of the OAT outcome measure, where stimulant use disorder is associated with significantly lower odds of OAT dispensation in the presence of concurrent mental illness (green rows), however this association does not hold in the absence of mental illness (yellow rows).

**Table S9. Mental illness and stimulant use disorder stratum specific unadjusted and adjusted Odds ratio estimates of OAT dispensation within two days of release among people with an opioid use disorder diagnosis who released from provincial prisons between Jan 1 2015- Dec 29 2018 (N=13,380) (P Value column included)**

|  | **Unadjusted**  **OR (95% CIs)** | **P value** | **Adjusted**  **OR (95% CIs)** | **P value** |
| --- | --- | --- | --- | --- |
| **No mental illness** |  |  |  |  |
| No stimulant use disorder | **0.66(0.58-0.76)** | **<0.001** | **0.64(0.57-0.74)** | **<0.001** |
| Stimulant use disorder | 0.93(0.77-1.18) | 0.533 | 0.89(0.70-1.13) | 0.344 |
| **Mental illness** |  |  |  |  |
| No stimulant use disorder | Reference | Reference | Reference | Reference |
| Stimulant use disorder | **0.81(0.70-0.92)** | **<0.001** | **0.73(0.64-0.84)** | **<0.001** |
| **Sex** |  |  |  |  |
| Female | **1.12(0.97-1.30)** | 0.132 | 1.16(0.99-1.35) | 0.056 |
| Male | Reference |  | Reference |  |
| **Age** |  |  |  |  |
| <30 | **0.64(0.47-0.86)** | 0.003 | **0.63(0.47-0.86)** | **0.003** |
| 30-39 | 0.75(0.56-1.01) | 0.058 | 0.75(0.56-1.02) | 0.07 |
| 40-49 | 0.97(0.63-1.18) | 0.368 | 0.90(0.66-1.24) | 0.533 |
| >=50 | Reference |  | Reference |  |
| **Health Authority** |  |  |  |  |
| Fraser | Reference |  | Reference |  |
| Interior | 1.44(1.24-1.69) | <0.001 | **1.31(1.12-1.54)** | 0.0006 |
| Vancouver Island | **1.70(1.45-2.00)** | <0.001 | **1.64(1.40-1.93)** | <0.001 |
| Northern | 0.94(0.74-1.18) | 0.566 | 0.87(0.69-1.10) | 0.253 |
| Vancouver Coastal | 1.10(0.95-1.27) | 0.210 | 1.08(0.93-1.25) | 0.309 |
| Unknown | **0.31(0.17-0.58)** | 0.0002 | **0.35(0.17-0.67)** | 0.002 |
| **Prior number of incarcerations (only back to 2015) at time of release** |  |  |  |  |
| 0 | **0.83(0.76-0.91)** | **<0.001** | 1.00(0.91-1.11) | 0.930 |
| 1 | **0.83(0.75-0.92)** | **0.002** | 0.95(0.86-1.05) | 0.299 |
| 2+ | Reference |  | Reference |  |
| **Year of release** |  |  |  |  |
| 2015 | Reference |  | Reference |  |
| 2016 | **1.31(1.14-1.51)** | 0.002 | **1.31(1.13-1.53)** | **0.003** |
| 2017 | **1.83(1.59-2.12)** | <0.001 | **1.86(1.59-2.16)** | **<0.001** |
| 2018 | **2.13(1.83-2.47)** | <0.001 | **2.15(1.82-2.52)** | **<0.001** |

**Footnote**: The Bonferroni adjustment was applied to adjust for multiple comparisons. The alpha was divided by the number of comparisons to determine the new p value for statistical significance. (0.05/7= 0.0071). The main associations of interest (Mental illness and Stimulant use disorder were statistically significant <0.001) and thus the conclusions of the analysis do not change.
